# Reliability of multimodal MRI brain measures in youth at risk for mental illness

**DOI:** 10.1002/brb3.1609

**Published:** 2020-04-18

**Authors:** Vladislav Drobinin, Holly Van Gestel, Carl A. Helmick, Matthias H. Schmidt, Chris V. Bowen, Rudolf Uher

**Affiliations:** ^1^ Department of Medical Neuroscience Dalhousie University Halifax NS Canada; ^2^ Nova Scotia Health Authority Halifax NS Canada; ^3^ Department of Psychiatry Dalhousie University Halifax NS Canada; ^4^ Department of Diagnostic Radiology Dalhousie University Halifax NS Canada

**Keywords:** developmental, diffusion tensor imaging, intraclass correlation coefficient, local gyrification index, MRI, reliability, reproducibility, youth

## Abstract

**Introduction:**

A new generation of large‐scale studies is using neuroimaging to investigate adolescent brain development across health and disease. However, imaging artifacts such as head motion remain a challenge and may be exacerbated in pediatric clinical samples. In this study, we assessed the scan–rescan reliability of multimodal MRI in a sample of youth enriched for risk of mental illness.

**Methods:**

We obtained repeated MRI scans, an average of 2.7 ± 1.4 weeks apart, from 50 youth (mean age 14.7 years, *SD* = 4.4). Half of the sample (52%) had a diagnosis of an anxiety disorder; 22% had attention‐deficit/hyperactivity disorder (ADHD). We quantified reliability with the test–retest intraclass correlation coefficient (ICC).

**Results:**

Gray matter measurements were highly reliable with mean ICCs as follows: cortical volume (ICC = 0.90), cortical surface area (ICC = 0.89), cortical thickness (ICC = 0.82), and local gyrification index (ICC = 0.85). White matter volume reliability was excellent (ICC = 0.98). Diffusion tensor imaging (DTI) components were also highly reliable. Fractional anisotropy was most consistently measured (ICC = 0.88), followed by radial diffusivity (ICC = 0.84), mean diffusivity (ICC = 0.81), and axial diffusivity (ICC = 0.78). We also observed regional variability in reconstruction, with some brain structures less reliably reconstructed than others.

**Conclusions:**

Overall, we showed that developmental MRI measures are highly reliable, even in youth at risk for mental illness and those already affected by anxiety and neurodevelopmental disorders. Yet, caution is warranted if patterns of results cluster within regions of lower reliability.

## Graphical abstract

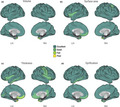

## 1 INTRODUCTION

A new generation of large‐scale studies (Alexander et al., 2017; Casey et al., 2018) is using neuroimaging techniques to investigate adolescent brain development across health and disease. These tremendous undertakings, often called “biobanks” for the wealth of biological data that they collect, are particularly focused on mental health. A primary goal includes identifying developmental trajectories of psychiatric illness which in turn might help improve early detection and guide intervention (Alexander et al., 2017; Casey et al., 2018). Such research is highly valuable, as epidemiologic studies show that 75% of psychiatric disorders begin early in the lifespan, prior to age 24 (Kessler et al., 2005; Kim‐Cohen et al., 2003). However, identifying clinically useful brain markers of illness, or “biomarkers,” hinges on the reliability of the MRI data.

Reliability is the ability of a measurement to provide consistent results under similar circumstances. Imaging artifacts, such as head motion, remain a challenge to reliability (Reuter et al., 2015), and there are concerns that measurement error may be exacerbated in pediatric clinical samples (Ducharme et al., 2016). Functional MRI studies have begun to address within‐subject reliability in youth as motion can have a profound effect on functional connectivity estimates (Van Dijk, Sabuncu, & Buckner, 2012; Vetter et al., 2017). However, a large body of imaging research deals with brain structure, and here too image artifacts are of concern. It has been shown that head motion in healthy volunteers can resemble cortical gray matter atrophy (Reuter et al., 2015). Children and adolescents might be particularly sensitive to scanner noise and may have difficulty remaining still for the duration of the sequences. One study examining pediatric MRI data has shown that low‐quality data can affect inferences regarding the developmental trajectories of cortical maturation (Ducharme et al., 2016). These findings necessitate the assessment of the reliability of MRI data in participants who are not merely undergoing normal development but are also showing externalizing and internalizing symptoms or are at increased familial risk for mental illness (Rasic, Hajek, Alda, & Uher, 2014).

In this study, we assessed the scan–rescan reliability of multimodal MRI in a sample of youth at risk for mental illness, including those already experiencing psychopathology. We measured common structural imaging metrics reported in the literature and quantified regional reliability based on widely used brain atlases. We also compared the reliability of structural measures to published estimates from samples of healthy adults.

## 2 MATERIALS AND METHODS

### 2.1 Participants

We recruited 53 youth (mean age 14.7 years, *SD* = 4.4) at familial risk for mental illness from FORBOW study, a longitudinal study enriched for sons and daughters of parents with mental illness (Uher et al., 2014). Offspring at familial risk for mental illness and participants from control families were invited to complete the MRI study. Participants were scanned twice, an average of 2.7 ± 1.4 weeks apart. Exclusion criteria were personal history of (i) psychotic illness, (ii) any serious medical or neurologic disorders, or (iii) MRI contraindications. The study protocol was approved by the Research Ethics Board of the Nova Scotia Health Authority. Participants provided written informed consent. For children who did not have capacity to make a fully informed decision, a parent or guardian provided written informed consent and the child provided assent.

### 2.2 Parent assessment

We used the Schedule for Affective Disorders and Schizophrenia (SADS‐IV; Endicott & Spitzer, 1978) and the Structured Clinical Interview for DSM‐5 (SCID‐5; First, 2015) to establish diagnoses of mental disorders according to DSM‐IV and DSM‐5.

### 2.3 Offspring assessment

Participating youth were interviewed using the Kiddie Schedule for Affective Disorders and Schizophrenia, Present and Lifetime Version (K‐SADS‐PL; Kaufman et al., 1997) by assessors blind to parent psychopathology. Diagnoses were confirmed in consensus meetings with a psychiatrist. Full‐scale intelligence quotient (FSIQ) was assessed using the Wechsler Abbreviated Scale of Intelligence (Wechsler, 2011).

### 2.4 Socioeconomic status (SES)

Socioeconomic status was captured as a composite variable (range 0–5) indexing: (i) maternal and (ii) paternal levels of education (iii) family household annual income, (iv) ownership of primary residence, and (v) ratio of bedrooms to residents in household, as previously described (MacKenzie et al., 2017; Zwicker et al., 2019). Higher numeric value reflects higher SES.

### 2.5 MRI acquisition

Images were acquired with a 3T General Electric Discovery MR750 scanner equipped with a 32‐channel MR Instruments RF head coil. Scanning took place at the Biomedical Translational Imaging Centre (BIOTIC), Halifax, Nova Scotia. Each participant was positioned supine in the MRI scanner with the head supported by foam padding to reduce movement. Earplugs were provided to minimize scanner noise. We collected a 3D T_1_‐weighted (T1w) Brain Volume imaging (BRAVO) sequence with whole‐brain coverage, 1 mm^3^ isotropic resolution, matrix = 224 × 224, field of view (FOV) = 224 mm, 168 sagittal slices at 1 mm thickness, repetition time (TR) = 5.9 ms, echo time (TE) = 2.2 ms, inversion time (TI) = 450 ms, flip angle = 12°, receiver bandwidth = ±62.5, number of excitations (NEX) = 2, autocalibrating reconstruction for cartesian imaging (ARC) phase acceleration = 2, ARC slice acceleration = 1, no phase wrap, scan duration = 5 min 42 s.

In addition, we collected a T_2_‐weighted fluid attenuated inversion recovery (FLAIR) sequence using a T2 prep contrast option (T2PREP) with identical coverage, resolution and acquisition orientation to the T1w sequence, TE = 98 ms, TR = 5,100 ms, TI = 1,427 ms, echo train length (ETL) = 250 echoes, flip angle = 90°, receiver bandwidth = ±62.5 kHz, NEX = 1, with prospective motion correction (PROMO) enabled (White et al., 2010), ARC phase = 2.5, ARC slice = 1, scan duration = 5 min.

Whole‐brain axial–oblique diffusion‐weighted images were also acquired using a single‐shot spin‐echo EPI pulse sequence, gradient directions = 30, *b*‐value = 1,000 s/mm^2^, three *b* = 0 images interleaved every 15 volumes, TR = 8,000 ms, TE = 66.7, FOV = 216 mm, slice thickness = 2 mm, number of slices 76, matrix = 108 × 108, voxel = 2^3^ mm isotropic, receiver bandwidth = ±250 kHz, ASSET phase acceleration factor = 2, phase‐encode direction = AP, scan duration = 4 min 32 s. For the purposes of estimating and correcting susceptibility‐induced distortions, we also acquired a second whole‐brain axial–oblique diffusion‐weighted sequence with matching parameters except only 8 volumes at *b* = 0 and with opposite phase‐encoding direction = PA, scan duration = 64 s.

### 2.6 MRI processing

Scans were processed with the Human Connectome Project (HCP) Minimal Preprocessing Pipeline (Glasser et al., 2013). The HCP pipeline is a well‐documented set of scripts developed to analyze high‐quality multimodal MRI data. It leverages the most widely used open‐source MRI processing software: FreeSurfer 6 (RRID:SCR_001847) (Fischl, 2012) and the FMRIB Software Library (FSL, RRID:SCR_002823) (Jenkinson, Beckmann, Behrens, Woolrich, & Smith, 2012). We have optimized the pipeline for our data by matching it to our acquisition parameters and by replacing the MNI template with a pediatric template for registration. The modified pipeline is available and freely accessible on https://github.com/GitDro/YouthReliability/tree/master/HCP_custom_pipeline. We used the NIHPD pediatric atlas (NIHPD Objective 1 atlases [4.5–18.5 years], RRID:SCR_008794) (Fonov et al., 2011) to minimize registration bias in our developmental cohort. In order to measure cortical folding, we ran the local gyrification index (LGI) analysis, the details of which can be found in the validation paper (Schaer et al., 2008) and on the https://surfer.nmr.mgh.harvard.edu/fswiki/LGI.

For gray matter reliability, we examined 34 cortical regions of interest per hemisphere based on the FreeSurfer default Desikan–Killiany atlas (Desikan et al., 2006). Thus, we measured cortical gray matter volume, cortical surface area, cortical thickness, and LGI/cortical folding in 68 parcellations per individual at each time point. Quality control was done both manually early in the processing stream and later with an automated supervised‐learning tool on the FreeSurfer segmented output. Manual quality ratings of T_1_‐weighted and T_2_‐weighted images were performed by authors VD and HVG. Automated quality control was done with the Qoala‐T tool (Klapwijk, Kamp, Meulen, Peters, & Wierenga, 2019). https://github.com/Qoala‐T/QC is an automated machine learning model used to classify the quality of FreeSurfer output. Six scans from three participants were excluded after the combined quality control (largely due to excess motion), bringing the total number of participants in the analysis to 50.

For white matter reliability, we examined white matter volume and diffusion tensor imaging (DTI) metrics based on the 20‐structure JHU DTI‐based white‐matter tractography atlas (Mori, Wakana, Zijl, & Nagae‐Poetscher, 2005). Data inclusion required absolute and relative motion to be under one and a half times the voxel size. Briefly, the processing was done in three steps: (i) creating binary maps of the 20 tracts in MNI152‐space, (ii) registering each binary map into subject diffusion–space by combining and applying the nonlinear warps from MNI152 to NIHPD space, and NIHPD space to subject T_1_‐weighted space, and the rigid‐body linear transform from subject T_1_‐weighted space to subject diffusion–space, (iii) using “fslstats” to report each metric; white matter volume, fractional anisotropy (FA), mean diffusivity (MD), axial diffusivity (AD), and radial diffusivity (RD) for each tract.

### 2.7 Statistical analysis

We used RStudio (R Version 3.6.2; RStudio version 1.2.5033; RStudio Team, 2019) to calculate the intraclass correlation coefficient (ICC) for the processed scan–rescan datasets. Reliability is the ability of a measurement to provide consistent results under similar circumstances. Test–retest reliability assesses stability under repeated tests, quantifying the extent to which measurements can be replicated. The ICC indexes both correlation and agreement between measurements (Koo & Li, 2016) and is commonly used to quantify reliability. We wanted to capture the variation in measurements taken by MRI and introduced in postprocessing, on the same participant under the same conditions weeks apart. We used ICC (1,1) for calculating scan–rescan reliability implemented in the https://cran.rproject.org/web/packages/ICC/index.html (Version 2.3.0; Wolak, Fairbairn, & Paulsen, 2012) which estimates the ICC and confidence intervals using the variance components form a one‐way ANOVA. We examined averaged ICC and the regional (parcellated) ICC for all measures and classified reliability according to generally defined criteria (Cicchetti, 1994): poor (<0.40), fair (0.41–0.59), good (>0.59–0.74), and excellent (>0.74). The code, data and analysis, is available https://github.com/GitDro/YouthReliability in a reproducible R notebook. We also repeated the analysis on a subsample of the participants scanned again twice, on average 14 months following their initial pair of scans (see Tables S1–S10).

## 3 RESULTS

### 3.1 Demographic and clinical characteristics

We present results from 100 scans collected from 50 youth (64% female) imaged several weeks apart (*M* = 2.70, *SD* = 1.36). The age range was 9–25 years old (*M* = 14.7, *SD* = 4.4). The majority of the participants have a family history of mental illness: 25 (50%) with a family history of major depressive disorder, 13 (26%) with a family history of bipolar disorder, and 2 (4%) with a family history of schizophrenia. Ten participants (20%) were recruited from control families. A large proportion of the scanned youth have been affected by mental illness: 26 participants (52%) had been diagnosed with an anxiety disorder, 13 (26%) had been diagnosed with major depressive disorder, and 11 (22%) have had a diagnosis of attention‐deficit/hyperactivity disorder (ADHD).

The sample was predominantly white (90%), with a minority (10%) comprised of indigenous and black youth. The composite SES indicator was normally distributed (*M* = 3.1, *SD* = 1.31). Full‐scale intelligence quotient (FSIQ) for the sample was in the normal range (*M* = 103, *SD* = 12.9).

### 3.2 Cortical volume

**FIGURE 1:**
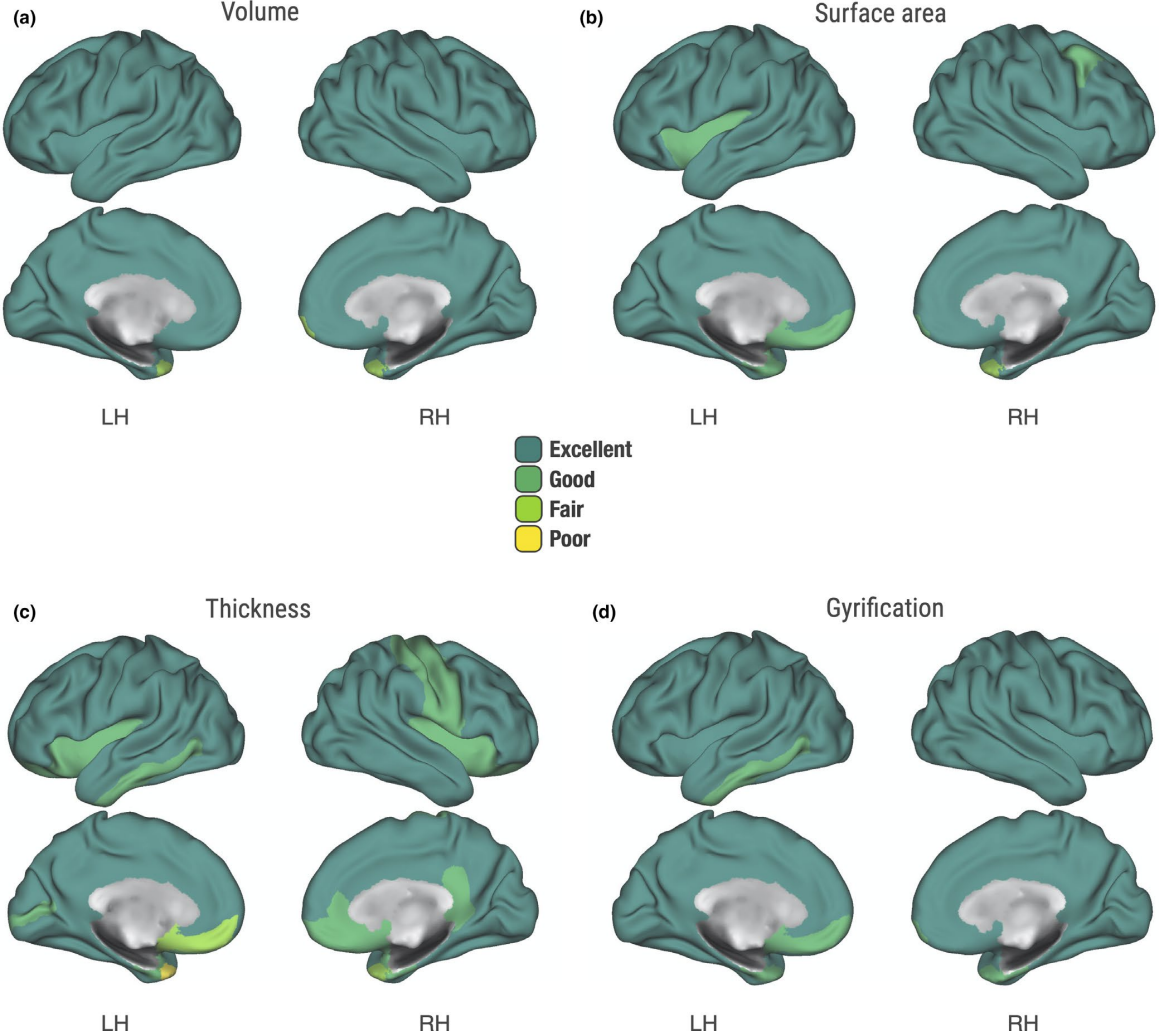
Reliability of cortical gray matter measures. Scan–rescan reliability of Desikan–Killiany regions. Intraclass correlation coefficient (ICC) values: poor (<0.40), fair (0.41–0.59), good (>0.59–0.74), and excellent (>0.74). (a) Cortical gray matter volume. (b) Cortical surface area. (c) Cortical thickness. (d) Local gyrification index

We observed “excellent” scan–rescan ICC for cortical gray matter volume (*M* = 0.90, 95% CI [0.84, 0.94]) averaged across the Desikan atlas regions (Figure 1a). The results were consistent across the left hemisphere (*M* = 0.92, 95% CI [0.86, 0.95]) and the right hemisphere (*M* = 0.89, 95% CI [0.82, 0.93]). As indicated by the high mean ICC, the reliability for most of the structures (65 out of 68; 96%) was classified as “excellent.” However, there was some regional variation (Table S1). The left supramarginal gyrus volume was the most reliably reconstructed, with near‐perfect ICC (ICC = 0.99, 95% CI [0.98, 0.99]). The volume of the left temporal pole was the least reliably measured (ICC = 0.47, 95% CI [0.23, 0.66]), with the ICC dipping into the “fair” classification and the lower bound of the confidence interval crossing the “poor” threshold. The contralateral right temporal pole was the next least reliably measured structure (ICC = 0.55, 95% CI [0.33, 0.72]). The only other structure with a designation below “excellent” was the right frontal pole, for which the ICC was only “fair” (ICC = 0.58, 95% CI [0.36, 0.74]). Cortical volume is a composite measure comprised of cortical surface area and cortical thickness; thus, we proceeded to examine the reliability of its components.

### 3.3 Cortical surface area

Averaged across the Desikan atlas regions, the ICC for cortical surface area (*M* = 0.89, 95% CI [0.82, 0.93]) was also deemed “excellent” overall (Figure 1b). Similar degree of reliability was attained both in the left hemisphere (*M* = 0.91, 95% CI [0.85, 0.95]) and the right hemisphere (*M* = 0.87, 95% CI [0.79, 0.92]). Just as with the volumetric results, the left supramarginal gyrus showed the highest ICC (ICC = 0.99, 95% CI [0.98, 0.99]). However, the ICCs for 12% of the Desikan regions were classified as “good” or “fair” (Table S2). The bilateral temporal poles were the least reliably reconstructed structures (left; ICC = 0.65, 95% CI [0.45, 0.78], right; ICC = 0.47, 95% CI [0.23, 0.66]). The frontal poles also showed lower ICCs than most structures (left; ICC = 0.69, 95% CI [0.51, 0.81], right; ICC = 0.70, 95% CI [0.53, 0.82]). The left insula, entorhinal cortex, and medial orbitofrontal cortex were classified as “good” with respective ICCs of 0.72, 0.71, 0.64, 95% CI [0.55, 0.83], [0.55, 0.83], [0.45, 0.78]. Finally, the right caudal middle frontal gyrus ICC confidence interval ranged from “fair” to “excellent” (ICC = 0.70, 95% CI [0.53, 0.82]).

### 3.4 Cortical thickness

Across the Desikan atlas, the mean ICC for cortical thickness was “good” to “excellent” (*M* = 0.82, 95% CI [0.71, 0.89]). The results were consistent across the left hemisphere (*M* = 0.83, 95% CI [0.73, 0.90]) and the right hemisphere (*M* = 0.81, 95% CI [0.69, 0.88]). The regional variability was more apparent than for other measures (Figure 1c), with 24% of the atlas below the “excellent” reliability designation (Table S3). Cortical thickness reconstruction was most reliable in the left superior frontal gyrus (ICC = 0.95, 95% CI [0.91, 0.97]). Once again, the temporal pole reconstruction was least reliable bilaterally (left; ICC = 0.38, 95% CI [0.12, 0.60], right; ICC = 0.41, 95% CI [0.16, 0.62]). Of note, two additional regions had the lower bound of the confidence interval cross into “poor” reliability. This included the right entorhinal cortex (ICC = 0.60, 95% CI [0.39, 0.75]) and the left medial orbitofrontal cortex (ICC = 0.56, 95% CI [0.34, 0.73]).

### 3.5 Cortical folding (LGI)

We also found “excellent” scan–rescan ICC (*M* = 0.85, 95% CI [0.75, 0.91]) for the measurement of cortical folding (Figure 1d). The average ICC across the right hemisphere was “excellent” (*M* = 0.85, 95% CI [0.76, 0.91]) and “good” to “excellent” across the left hemisphere (*M* = 0.84, 95% CI [0.74, 0.90]). Regional reliability was fairly consistent, with most structures displaying “excellent” reconstruction, and the rest (eight structures) achieving “good” ICCs. None of the confidence intervals dipped into the “poor” classification (Table S4). Cortical folding reconstruction was most reliable in the right precentral gyrus (ICC = 0.95, 95% CI [0.92, 0.97]). As expected by now, the bilateral frontal (left; ICC = 0.62, 95% CI [0.41, 0.76], right; ICC = 0.66, 95% CI [0.48, 0.79]) and temporal poles (left; ICC = 0.63, 95% CI [0.43, 0.77], right; ICC = 0.68 95% CI [0.50, 0.81]) had the comparably lowest ICC estimates in measures of regional gyrification. The gyrification measurement of entorhinal cortex was also among the least reliable (left; ICC = 0.65, 95% CI [0.45, 0.78], right; ICC = 0.71, 95% CI [0.54, 0.82]), however, still “fair” to “excellent.”

### 3.6 White matter volume

We observed remarkable reliability of white matter volume measurements averaged across the JHU white‐matter tractography atlas (*M* = 0.98, 95% CI [0.97, 0.99]). Near‐perfect reliability was observed in the reconstruction of the cingulum near the cingulate gyrus (ICC = 0.99, 95% CI [0.99, 1.00]) and other white matter tracts (Table S5). The lowest regional reliability was observed in the cingulum near the hippocampus (ICC = 0.96, 95% CI [0.93, 0.98]), which was nevertheless categorized as “excellent.”

### 3.7 Diffusion tensor imaging (DTI) measures

#### 3.7.1 Fractional anisotropy (FA)

**FIGURE 2:**
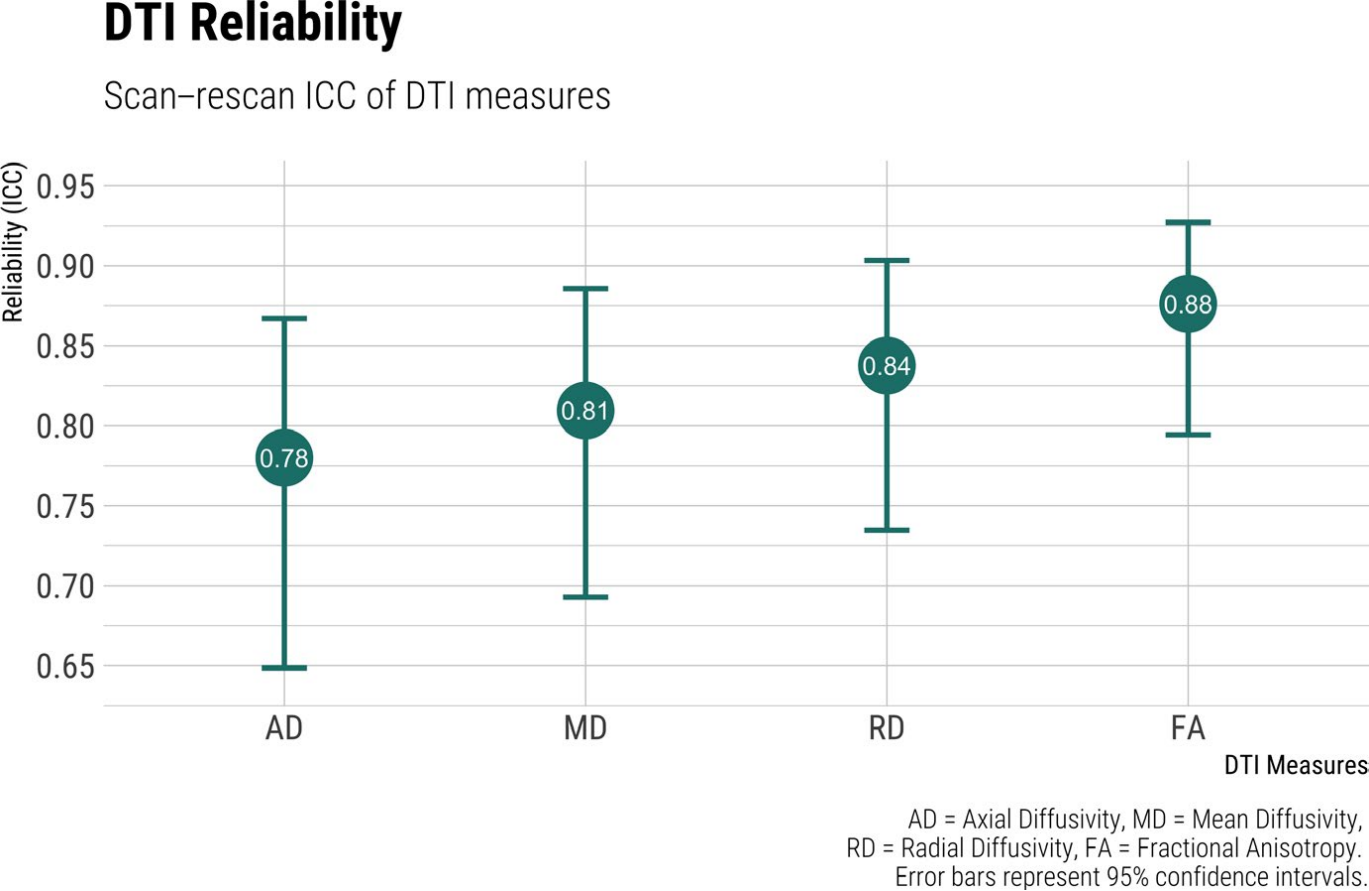
Scan–rescan reliability of diffusion tensor imaging (DTI) measures

Next, we examined white matter FA, which is often used to index microstructural integrity. Scan–rescan ICCs were “excellent” averaged across the JHU atlas (*M* = 0.88, 95% CI [0.79, 0.93]), Figure 2. The left superior longitudinal fasciculus was measured most reliably across scan sessions (ICC = 0.95, 95% CI [0.91, 0.97]). The forceps minor, also known as the anterior forceps, runs bilaterally and had the lowest reliability estimates for FA (ICC = 0.76, 95% CI [0.61, 0.86]). The test–retest ICCs for all regions of the atlas fell into the “excellent” range; however, a number of the regions had the lower confidence interval overlap with the “good” threshold (Table S6).

#### 3.7.2 Radial diffusivity (RD)

Radial diffusivity has been previously used as a proxy measure for myelin damage or demyelination. In our study, we found the measure to be, on average, of “good” to “excellent” reliability (*M* = 0.84, 95% CI [0.73, 0.90]). The forceps major, also known as the posterior forceps, was the white matter fiber bundle with the highest RD reliability (ICC = 0.94, 95% CI [0.90, 0.97]). The right hippocampal cingulum bundle had the lowest scan–rescan reliability (ICC = 0.69, 95% CI [0.52, 0.81]). Of note, the lower confidence interval around the ICC was “fair” for five white matter tracts (Table S7).

#### 3.7.3 Mean diffusivity (MD)

Mean diffusivity summarizes the average diffusion properties of a voxel and can be sensitive to pathology such as edema and necrosis, among others. Overall, mean atlas‐averaged ICCs were “good” to “excellent” (*M* = 0.81, 95% CI [0.69, 0.89]). Based on the lower CI bounds, six white matter tracts overlap with the “fair” reliability classification (Table S8). Among those are the bilateral cingulum bundles surrounding the hippocampus (left; ICC = 0.65, 95% CI [0.46, 0.79], right; ICC = 0.63, 95% CI [0.43, 0.77]) and the corticospinal tract (left; ICC = 0.69, 95% ICC [0.52, 0.81], right; ICC = 0.66, 95% CI [0.48, 0.79]). Once again, the highest scan–rescan reliability was observed in the measurement of the forceps minor (ICC = 0.91, 95% CI [0.85, 0.95]) and forceps major (ICC = 0.93, 95% CI [0.89, 0.96]).

#### 3.7.4 Axial diffusivity (AD)

Axial diffusivity measures water diffusion along the principal axis of diffusion and may be correlated with axonal injury. AD had the lowest average ICC of the DTI scalars in our study (*M* = 0.78, 95% CI [0.65, 0.87]). While the overall ICC can be classified as “good” to “excellent,” there is some regional variability of note (Table S9). The right hippocampal cingulum bundle had the lowest scan–rescan ICC, with the lower confidence interval crossing into the “poor” classification (ICC = 0.53, 95% CI [0.30, 0.70]). Nevertheless, some regions stood out for their excellent scan–rescan reliability, such as the forceps major (ICC = 0.93, 95% CI [0.88, 0.96]) and the bilateral anterior thalamic radiation (left; ICC = 0.90, 95% CI [0.84, 0.94], right; ICC = 0.90, 95% CI [0.83, 0.94]).

## 4 DISCUSSION

In this paper, we report the reliability of nine MRI‐derived measures of cortical and white matter morphology and integrity based on 100 scans from 50 youth. Despite the high prevalence of anxiety and ADHD disorders in our young sample, we found good to excellent reliability for all measures. White matter volume was most consistently reconstructed with a scan–rescan ICC of 0.98 averaged across the white matter atlas. Axial diffusivity was the least reliable, with an average ICC of 0.78 across scan sessions. We also observed regional variability in reconstruction, with many structures showing excellent stability across measures, and some showing poor to fair reconstruction. This analysis might be of particular interest for hypothesis driven studies focusing on select regions of interest, and for exploratory and predictive multivariate studies to cross reference the pattern of findings to their reported reliability distributions.

The excellent reliability of gray matter measures should be interpreted in the context of prior work. While the reliability of functional MRI data in youth has received some attention (Thomason et al., 2011; Vetter et al., 2017), literature examining the reliability of structural MRI data remains sparse. Therefore, we interpret the consistency of our data by comparing it to similar work in adult samples. Iscan and colleagues (Iscan et al., 2015) reported a comparable analysis to ours. Their study included 40 healthy controls (age 18–65), scanned twice, whose MRI images were processed in FreeSurfer. Overall, 25 individuals passed their thorough quality control. In the approved scans, reported ICCs for cortical thickness/ surface area/ volume were 0.81, 0.87, and 0.88; remarkably similar to our values of 0.82, 0.89, and 0.90. The closeness of these values carries two messages: (i) It is possible to collect highly reliable MRI data from young people with anxiety and/or ADHD, and (ii) after proper quality control, the reliability can compare to that attained from scans of healthy adults.

We extended our gray matter analysis to investigate the reliability of cortical folding (LGI), an important neurodevelopmental marker that is essential to the optimization of axonal wiring and the functional organization of the brain (Klyachko & Stevens, 2003). With an ICC of 0.85, cortical folding was of excellent reliability, ranking between measures of cortical thickness and cortical surface area. Cortical folding reliability was slightly lower than what was reported (ICC = 0.94) in a recent paper (Madan & Kensinger, 2017). The difference can be attributed to several factors, as the prior work focused on healthy adults who were scanned either 10 times or with a sequence specifically optimized for brain morphology research. To our knowledge, the current study is the first to report on the reliability of this morphological measure in a pediatric risk sample.

Out of all the cortical gray matter measures, cortical thickness had the lowest ICC overall and had the most structures categorized to be of poor reliability based on their lower bound confidence interval. The average thickness of the cortical mantle is 2.5 mm (Fischl & Dale, 2000) which is close to the 1 mm spatial resolution of most scan sequences. Thereby cortical thickness measurements may be particularly sensitive to motion artifacts even in high‐quality data (Alexander‐Bloch et al., 2016).

The structures with the least reliable cortical thickness reconstructions were the temporal pole, frontal pole, medial orbitofrontal gyrus, and the entorhinal cortex. The temporal and frontal poles also exhibited reduced reconstruction consistency in analysis of gray matter volume and surface area, and are known to be problematic in the literature (Klapwijk et al., 2019). The medial orbitofrontal gyrus and entorhinal cortex are localized to the inferior aspect of the brain, and their location makes them particularly affected by susceptibility gradients from air‐filled cavities, the bone–tissue interface, and orbital artifacts. However, the orbitofrontal and entorhinal cortices are both essential to fundamental aspects of memory and cognition and have been implicated in a wide range of disorders (Baiano et al., 2008; Rolls & Grabenhorst, 2008). Our results suggest the need for stringent quality control and adequately powered samples in future studies of the cortical thickness of these areas.

In contrast, white matter volume had the highest reconstruction reliability in our study. The near‐perfect ICC, both regionally and overall, makes the measure particularly suitable for longitudinal research. However, the assessment of white matter microstructure with diffusion tensor imaging (DTI) was more variable. DTI is widely used to infer white matter microstructure, structural connectivity, and axonal health. Our results ranged from good to excellent (ICC 0.78–0.88) for the four DTI measures, with axial diffusivity (AD) being the least reliable and fractional anisotropy (FA) the most reliable. This mirrors the relative interest attained for these measures in the research community. AD may be a correlate of axonal injury (Budde, Xie, Cross, & Song, 2009); however, the measure is less widely used than FA which has been the most popular correlate of white matter integrity (Soares, Marques, Alves, & Sousa, 2013). Regionally, none of the lower confidence intervals for FA ICCs crossed below the good into the fair or poor classification.

Across all DTI measures, the only region with the lower bound confidence interval in the poor classification was the hippocampal cingulum bundle. This white matter tract, along with the cingulum cingulate bundle, had the lowest scan–rescan reliability estimates for AD, MD, and RD. The cingulum bundle is a large white matter tract interconnecting the frontal, parietal, medial temporal, and other areas and has been implicated in a spectrum of neuropsychiatric disorders (Bubb, Metzler‐Baddeley, & Aggleton, 2018). Its size and midline positioning might make it particularly susceptible to motion artifacts and spatial misregistration errors, and thus, a similar warning akin to low reliability areas of cortical thickness applies here as well.

Lower scan–rescan reliability also applied to the corticospinal tract. Interpreting these findings in the context of prior research might be illuminating. Investigations of the underlying reliability of white matter measures in pediatric samples have mainly been restricted to small samples, specific illness, or a limited number of white matter tracts (Alhamud, Taylor, Laughton, Kouwe, & Meintjes, 2015; Bonekamp et al., 2007; Carlson et al., 2014). However, a recent paper has examined FA reliability in a well‐powered sample comprising of both an adult and an adolescent group (Acheson et al., 2017). Similar to our results, the authors found that in adolescents, the lowest reliability was observed in the corticospinal tract. This observation held in adults, signifying low reliability of the corticospinal tract across development. The corticospinal tract is a white matter motor pathway, and thus, the reliability concerns might not be immediately relevant to psychiatric research.

Lastly, our structural MRI reliability estimates were higher than those reported in functional MRI literature. An early account provided the first empirical evidence of the longitudinal reliability of resting state fMRI in children (Thomason et al., 2011). The authors obtained positive ICC values for the majority of brain voxels, indicating stability within participants across measurements. The first group to investigate the reliability of resting state fMRI in clinical developing groups observed fair (>0.40) to good (>0.70) ICC in the short term (Somandepalli et al., 2015). The authors noted higher ICC in typically developing children compared to those with ADHD. A more recent report examined reliability in adolescent fMRI within a 2‐year period (Vetter et al., 2017). The investigators found both variability and stability, with the reliability results dependent on task domain and region of interest. For example, whole‐brain ICC was lower (0.44) in cognitive control paradigms and higher (0.74) in reward paradigms. There was great variability across regions of interest, with ICCs ranging from poor (0.19) to excellent (0.84). Two recent meta‐analyses suggest that even these modest fMRI reliability values are potentially optimistic.

One meta‐analysis examined a decade of test–retest reliability work surrounding functional connectivity. The authors concluded that most functional connections exhibited “poor” ICC of 0.29 (95% CI [0.23, 0.36]; Noble, Scheinost, & Constable, 2019). Another recent meta‐analysis examined test–retest reliability of common task‐based fMRI measures (Elliott et al., 2020). Echoing the previously mentioned findings, their work revealed poor to fair overall reliability (ICC = 0.40) across 90 studies. However, it is worth noting that contrasting the reliability of structural and functional MRI is not an apples‐to‐apples comparison. The excellent structural reliability we report in this manuscript is based on the consistent reconstruction of a priori anatomically defined regions. Functional reliability deals with spatial, temporal, and frequency domains that often try to map onto fluid brain processes. Nevertheless, the discrepancy between the two modalities is worth acknowledging as it can have practical applications, such as sample size requirements for biomarker discovery (Elliott et al., 2020).

### 4.1 Limitations

There are several limitations to this study. Sample size is of concern, not in respect to accurately estimating reliability but to problems of scale. Our approach of manual ratings for raw data followed by automated quality assessment for processed data can become resource intensive for large‐scale projects, such as the modern biobanks collecting tens of thousands of scans. Our relatively small number of excluded scans would grow substantially in those samples and could potentially vary between groups of interest, for example, those with or without psychopathology. Nevertheless, this is actively being addressed with behavioral interventions before or during scanning, with optimized sequences utilizing prospective motion correction, as well as at the study design phase with oversampling of at‐risk youth.

We were also restricted to a single scan site, and data were acquired on the same scanner at all time points. In our study, we found that results generalized to the same scanner over a year later (Table S10). However, large collaborative efforts are often made possible by acquisitions on scanners from different manufacturers at sites that may be continents apart (Thompson et al., 2014). This can increase variability that confounds the effects of interest. Nevertheless, these challenges are being overcome with the standardization of scanning parameters and statistical techniques that correct for site differences (Chen et al., 2014). Lastly, beyond site differences, variations in data analysis methods are more likely to have a stronger effect on neuroimaging results, but are also being addressed (Nichols et al., 2017).

Another limitation is our choice of parcellation scheme for assessing regional reliability of cortical areas and white matter tracts. The construction of an accurate map of the major subdivisions of the human brain is a century‐old endeavor with an accompanying and equally long debate on what constitutes a boundary. There are other parcellations than the one used in this paper that are more biologically grounded, accounting for cortical architecture, topography, and functional connectivity (Glasser et al., 2016). However, given that it is impossible to exhaustively test each parcellation, we decided to focus on those most likely to be commonly used in the field. The Desikan atlas has over 5,000 citations on PubMed, and the JHU atlas has almost 2,000. They come default or preinstalled with commonly used MRI software, including FreeSurfer and FSL, respectively. Thus, these atlases are the starting point for a great number of neuroimaging researchers and a basis of comparison for those on the cutting edge who choose to use newer or custom parcellations.

## 5 CONCLUSIONS

In conclusion, while researchers should be cognizant of regional variability in reconstruction, pediatric MRI brain data are highly reliable overall. Furthermore, the high reliability was established in youth at risk for mental illness or those already affected by anxiety and neurodevelopmental disorders. This bodes well for work investigating the neurodevelopmental markers of mental illness at an early stage, before medication, drug use, and other confounds take a persistent toll on the brain. Confidence in the data quality of high‐risk youth samples is also a prerequisite for improved diagnosis and development of personalized prevention strategies based on brain markers.

## CONFLICT OF INTEREST

None of the authors have any conflicts of interest to declare.

## AUTHOR CONTRIBUTIONS

V. Drobinin, M. Schmidt, C. Bowen, and R. Uher designed the study. V. Drobinin, H. Van Gestel, C. Helmick, and R. Uher acquired the data, which V. Drobinin and R. Uher analyzed. V. Drobinin wrote the article, which all authors reviewed. All authors approved the final version to be published and can certify that no other individuals not listed as authors have made substantial contributions to the paper.

## Supporting information

Table S1‐S10

## Data Availability

The data that support the findings of this study are openly available in Zenodo at https://doi.org/10.5281/zenodo.3627320 (Drobinin, 2020).
