# Supplementary material for: Reliability of multimodal MRI brain measures in youth at risk for mental illness
Source: Brain Behav. 2020 Apr 18;10(6):e01609. doi: 10.1002/brb3.1609 (PMC7303399; doi:10.1002/brb3.1609)
Supplement: Supplementary file 1 — Table S1‐S10 [file BRB3-10-e01609-s001.docx]

**Supplement Table 1. Grey matter volume reliability**

|  | | **Left Hemisphere** | | | **Right Hemisphere** | | |
| --- | --- | --- | --- | --- | --- | --- | --- |
| **ROI** | **Overall ICC** | **2.5% CI** | **ICC** | **97.5% CI** | **2.5% CI** | **ICC** | **97.5% CI** |
| Banks of the Superior Temporal Sulcus | 0.95 | 0.93 | 0.96 | 0.98 | 0.91 | 0.95 | 0.97 |
| Caudal Anterior Cingulate | 0.86 | 0.71 | 0.82 | 0.89 | 0.84 | 0.91 | 0.95 |
| Caudal Middle Frontal | 0.90 | 0.94 | 0.97 | 0.98 | 0.72 | 0.83 | 0.90 |
| Cuneus | 0.96 | 0.92 | 0.95 | 0.97 | 0.95 | 0.97 | 0.98 |
| Entorhinal | 0.79 | 0.72 | 0.83 | 0.90 | 0.60 | 0.75 | 0.85 |
| Fusiform | 0.96 | 0.90 | 0.94 | 0.97 | 0.95 | 0.97 | 0.98 |
| Inferior Parietal | 0.97 | 0.93 | 0.96 | 0.98 | 0.96 | 0.98 | 0.99 |
| Inferior Temporal | 0.98 | 0.97 | 0.98 | 0.99 | 0.95 | 0.97 | 0.98 |
| Isthmus Cingulate | 0.93 | 0.92 | 0.95 | 0.97 | 0.85 | 0.91 | 0.95 |
| Lateral Occipital | 0.96 | 0.91 | 0.95 | 0.97 | 0.94 | 0.97 | 0.98 |
| Lateral Orbitofrontal | 0.87 | 0.82 | 0.89 | 0.94 | 0.75 | 0.85 | 0.91 |
| Lingual | 0.97 | 0.96 | 0.97 | 0.99 | 0.92 | 0.96 | 0.97 |
| Medial Orbitofrontal | 0.88 | 0.70 | 0.82 | 0.89 | 0.89 | 0.94 | 0.96 |
| Middle Temporal | 0.96 | 0.95 | 0.97 | 0.98 | 0.93 | 0.96 | 0.98 |
| Parahippocampal | 0.90 | 0.85 | 0.91 | 0.95 | 0.80 | 0.88 | 0.93 |
| Paracentral | 0.93 | 0.92 | 0.95 | 0.97 | 0.84 | 0.91 | 0.94 |
| Pars Opercularis | 0.90 | 0.95 | 0.97 | 0.98 | 0.72 | 0.83 | 0.90 |
| Pars Orbitalis | 0.85 | 0.68 | 0.81 | 0.89 | 0.82 | 0.89 | 0.94 |
| Pars Triangularis | 0.94 | 0.92 | 0.95 | 0.97 | 0.86 | 0.92 | 0.95 |
| Pericalcarine | 0.95 | 0.91 | 0.94 | 0.97 | 0.91 | 0.95 | 0.97 |
| Postcentral | 0.96 | 0.97 | 0.98 | 0.99 | 0.88 | 0.93 | 0.96 |
| Posterior Cingulate | 0.93 | 0.88 | 0.93 | 0.96 | 0.88 | 0.93 | 0.96 |
| Precentral | 0.94 | 0.91 | 0.95 | 0.97 | 0.90 | 0.94 | 0.96 |
| Precuneus | 0.93 | 0.97 | 0.99 | 0.99 | 0.79 | 0.87 | 0.93 |
| Rostral Anterior Cingulate | 0.93 | 0.89 | 0.94 | 0.96 | 0.86 | 0.92 | 0.95 |
| Rostral Middle Frontal | 0.94 | 0.95 | 0.97 | 0.98 | 0.84 | 0.91 | 0.95 |
| Superior Frontal | 0.94 | 0.97 | 0.98 | 0.99 | 0.82 | 0.89 | 0.94 |
| Superior Parietal | 0.91 | 0.96 | 0.98 | 0.99 | 0.72 | 0.83 | 0.90 |
| Superior Temporal | 0.97 | 0.96 | 0.98 | 0.99 | 0.94 | 0.96 | 0.98 |
| Supramarginal | 0.92 | 0.98 | 0.99 | 0.99 | 0.76 | 0.86 | 0.92 |
| Frontal Pole | 0.68 | 0.64 | 0.78 | 0.87 | 0.36 | 0.58 | 0.74 |
| Temporal Pole | 0.51 | 0.23 | 0.47 | 0.66 | 0.33 | 0.55 | 0.72 |
| Transverse Temporal | 0.90 | 0.84 | 0.91 | 0.95 | 0.82 | 0.89 | 0.94 |
| Insula | 0.87 | 0.75 | 0.85 | 0.91 | 0.83 | 0.90 | 0.94 |

**Supplement Table 2. Cortical surface area reliability**

|  | | **Left Hemisphere** | | | **Right Hemisphere** | | |
| --- | --- | --- | --- | --- | --- | --- | --- |
| **ROI** | **Overall ICC** | **2.5% CI** | **ICC** | **97.5% CI** | **2.5% CI** | **ICC** | **97.5% CI** |
| Banks of the Superior Temporal Sulcus | 0.95 | 0.92 | 0.96 | 0.97 | 0.91 | 0.94 | 0.97 |
| Caudal Anterior Cingulate | 0.89 | 0.80 | 0.88 | 0.93 | 0.83 | 0.90 | 0.94 |
| Caudal Middle Frontal | 0.84 | 0.97 | 0.98 | 0.99 | 0.53 | 0.70 | 0.82 |
| Cuneus | 0.96 | 0.92 | 0.95 | 0.97 | 0.94 | 0.96 | 0.98 |
| Entorhinal | 0.74 | 0.55 | 0.71 | 0.83 | 0.62 | 0.77 | 0.86 |
| Fusiform | 0.95 | 0.87 | 0.93 | 0.96 | 0.95 | 0.97 | 0.98 |
| Inferior Parietal | 0.97 | 0.93 | 0.96 | 0.98 | 0.96 | 0.98 | 0.99 |
| Inferior Temporal | 0.95 | 0.91 | 0.95 | 0.97 | 0.92 | 0.96 | 0.97 |
| Isthmus Cingulate | 0.93 | 0.90 | 0.94 | 0.97 | 0.86 | 0.92 | 0.95 |
| Lateral Occipital | 0.97 | 0.92 | 0.96 | 0.97 | 0.97 | 0.98 | 0.99 |
| Lateral Orbitofrontal | 0.81 | 0.70 | 0.82 | 0.89 | 0.67 | 0.80 | 0.88 |
| Lingual | 0.96 | 0.94 | 0.97 | 0.98 | 0.91 | 0.95 | 0.97 |
| Medial Orbitofrontal | 0.77 | 0.45 | 0.64 | 0.78 | 0.82 | 0.89 | 0.94 |
| Middle Temporal | 0.97 | 0.95 | 0.97 | 0.98 | 0.94 | 0.97 | 0.98 |
| Parahippocampal | 0.88 | 0.84 | 0.91 | 0.95 | 0.77 | 0.86 | 0.92 |
| Paracentral | 0.95 | 0.93 | 0.96 | 0.98 | 0.91 | 0.94 | 0.97 |
| Pars Opercularis | 0.93 | 0.93 | 0.96 | 0.98 | 0.83 | 0.90 | 0.94 |
| Pars Orbitalis | 0.90 | 0.86 | 0.92 | 0.95 | 0.79 | 0.88 | 0.93 |
| Pars Triangularis | 0.92 | 0.92 | 0.95 | 0.97 | 0.81 | 0.89 | 0.93 |
| Pericalcarine | 0.94 | 0.87 | 0.92 | 0.95 | 0.93 | 0.96 | 0.98 |
| Postcentral | 0.91 | 0.98 | 0.99 | 0.99 | 0.73 | 0.84 | 0.90 |
| Posterior Cingulate | 0.90 | 0.82 | 0.89 | 0.94 | 0.85 | 0.91 | 0.95 |
| Precentral | 0.86 | 0.94 | 0.97 | 0.98 | 0.61 | 0.76 | 0.85 |
| Precuneus | 0.93 | 0.98 | 0.99 | 0.99 | 0.79 | 0.88 | 0.93 |
| Rostral Anterior Cingulate | 0.89 | 0.84 | 0.91 | 0.95 | 0.78 | 0.87 | 0.92 |
| Rostral Middle Frontal | 0.91 | 0.94 | 0.97 | 0.98 | 0.76 | 0.86 | 0.92 |
| Superior Frontal | 0.89 | 0.95 | 0.97 | 0.98 | 0.69 | 0.81 | 0.89 |
| Superior Parietal | 0.89 | 0.98 | 0.99 | 0.99 | 0.67 | 0.80 | 0.88 |
| Superior Temporal | 0.98 | 0.97 | 0.98 | 0.99 | 0.95 | 0.97 | 0.98 |
| Supramarginal | 0.92 | 0.98 | 0.99 | 0.99 | 0.74 | 0.84 | 0.91 |
| Frontal Pole | 0.69 | 0.51 | 0.69 | 0.81 | 0.53 | 0.70 | 0.82 |
| Temporal Pole | 0.56 | 0.45 | 0.65 | 0.78 | 0.23 | 0.47 | 0.66 |
| Transverse Temporal | 0.93 | 0.88 | 0.93 | 0.96 | 0.87 | 0.92 | 0.96 |
| Insula | 0.74 | 0.55 | 0.72 | 0.83 | 0.62 | 0.76 | 0.86 |

**Supplement Table 3. Cortical thickness reliability**

|  | | **Left Hemisphere** | | | **Right Hemisphere** | | |
| --- | --- | --- | --- | --- | --- | --- | --- |
| **ROI** | **Overall ICC** | **2.5% CI** | **ICC** | **97.5% CI** | **2.5% CI** | **ICC** | **97.5% CI** |
| Banks of the Superior Temporal Sulcus | 0.93 | 0.91 | 0.95 | 0.97 | 0.86 | 0.92 | 0.95 |
| Caudal Anterior Cingulate | 0.81 | 0.65 | 0.78 | 0.87 | 0.72 | 0.83 | 0.90 |
| Caudal Middle Frontal | 0.91 | 0.85 | 0.91 | 0.95 | 0.85 | 0.91 | 0.95 |
| Cuneus | 0.89 | 0.81 | 0.89 | 0.94 | 0.80 | 0.88 | 0.93 |
| Entorhinal | 0.61 | 0.43 | 0.63 | 0.77 | 0.39 | 0.60 | 0.75 |
| Fusiform | 0.87 | 0.83 | 0.90 | 0.94 | 0.73 | 0.84 | 0.90 |
| Inferior Parietal | 0.93 | 0.89 | 0.94 | 0.96 | 0.88 | 0.93 | 0.96 |
| Inferior Temporal | 0.87 | 0.76 | 0.85 | 0.91 | 0.80 | 0.88 | 0.93 |
| Isthmus Cingulate | 0.75 | 0.64 | 0.78 | 0.87 | 0.55 | 0.71 | 0.83 |
| Lateral Occipital | 0.87 | 0.79 | 0.87 | 0.93 | 0.76 | 0.86 | 0.92 |
| Lateral Orbitofrontal | 0.68 | 0.50 | 0.68 | 0.81 | 0.49 | 0.67 | 0.80 |
| Lingual | 0.84 | 0.73 | 0.83 | 0.90 | 0.76 | 0.85 | 0.91 |
| Medial Orbitofrontal | 0.62 | 0.34 | 0.56 | 0.73 | 0.48 | 0.67 | 0.80 |
| Middle Temporal | 0.85 | 0.81 | 0.89 | 0.93 | 0.68 | 0.81 | 0.89 |
| Parahippocampal | 0.88 | 0.79 | 0.88 | 0.93 | 0.79 | 0.88 | 0.93 |
| Paracentral | 0.86 | 0.79 | 0.88 | 0.93 | 0.76 | 0.85 | 0.91 |
| Pars Opercularis | 0.90 | 0.87 | 0.92 | 0.96 | 0.78 | 0.87 | 0.92 |
| Pars Orbitalis | 0.75 | 0.60 | 0.75 | 0.85 | 0.60 | 0.75 | 0.85 |
| Pars Triangularis | 0.92 | 0.87 | 0.92 | 0.95 | 0.87 | 0.92 | 0.95 |
| Pericalcarine | 0.73 | 0.54 | 0.71 | 0.83 | 0.60 | 0.75 | 0.85 |
| Postcentral | 0.80 | 0.91 | 0.95 | 0.97 | 0.47 | 0.66 | 0.79 |
| Posterior Cingulate | 0.87 | 0.76 | 0.85 | 0.91 | 0.80 | 0.88 | 0.93 |
| Precentral | 0.82 | 0.85 | 0.91 | 0.95 | 0.56 | 0.73 | 0.83 |
| Precuneus | 0.93 | 0.88 | 0.93 | 0.96 | 0.87 | 0.92 | 0.96 |
| Rostral Anterior Cingulate | 0.74 | 0.60 | 0.75 | 0.85 | 0.56 | 0.72 | 0.83 |
| Rostral Middle Frontal | 0.93 | 0.85 | 0.91 | 0.95 | 0.91 | 0.95 | 0.97 |
| Superior Frontal | 0.95 | 0.91 | 0.95 | 0.97 | 0.90 | 0.94 | 0.97 |
| Superior Parietal | 0.92 | 0.89 | 0.94 | 0.96 | 0.84 | 0.90 | 0.94 |
| Superior Temporal | 0.92 | 0.87 | 0.92 | 0.96 | 0.85 | 0.91 | 0.95 |
| Supramarginal | 0.93 | 0.90 | 0.94 | 0.97 | 0.85 | 0.91 | 0.95 |
| Frontal Pole | 0.69 | 0.62 | 0.76 | 0.86 | 0.42 | 0.62 | 0.77 |
| Temporal Pole | 0.40 | 0.12 | 0.38 | 0.60 | 0.16 | 0.41 | 0.62 |
| Transverse Temporal | 0.82 | 0.71 | 0.82 | 0.90 | 0.71 | 0.83 | 0.90 |
| Insula | 0.63 | 0.46 | 0.66 | 0.79 | 0.41 | 0.61 | 0.76 |

**Supplement Table 4. Local gyrification index reliability**

|  | | **Left Hemisphere** | | | **Right Hemisphere** | | |
| --- | --- | --- | --- | --- | --- | --- | --- |
| **ROI** | **Overall ICC** | **2.5% CI** | **ICC** | **97.5% CI** | **2.5% CI** | **ICC** | **97.5% CI** |
| Banks of the Superior Temporal Sulcus | 0.85 | 0.68 | 0.81 | 0.89 | 0.81 | 0.89 | 0.94 |
| Caudal Anterior Cingulate | 0.82 | 0.68 | 0.81 | 0.89 | 0.73 | 0.84 | 0.91 |
| Caudal Middle Frontal | 0.93 | 0.91 | 0.95 | 0.97 | 0.87 | 0.92 | 0.96 |
| Cuneus | 0.90 | 0.87 | 0.92 | 0.95 | 0.79 | 0.87 | 0.93 |
| Entorhinal | 0.68 | 0.45 | 0.65 | 0.78 | 0.54 | 0.71 | 0.82 |
| Fusiform | 0.83 | 0.65 | 0.79 | 0.87 | 0.80 | 0.88 | 0.93 |
| Inferior Parietal | 0.92 | 0.82 | 0.90 | 0.94 | 0.91 | 0.95 | 0.97 |
| Inferior Temporal | 0.78 | 0.53 | 0.70 | 0.82 | 0.76 | 0.86 | 0.92 |
| Isthmus Cingulate | 0.83 | 0.70 | 0.82 | 0.89 | 0.76 | 0.85 | 0.91 |
| Lateral Occipital | 0.92 | 0.84 | 0.90 | 0.94 | 0.89 | 0.94 | 0.96 |
| Lateral Orbitofrontal | 0.79 | 0.65 | 0.78 | 0.87 | 0.66 | 0.79 | 0.88 |
| Lingual | 0.89 | 0.81 | 0.89 | 0.93 | 0.83 | 0.90 | 0.94 |
| Medial Orbitofrontal | 0.73 | 0.54 | 0.71 | 0.82 | 0.59 | 0.75 | 0.85 |
| Middle Temporal | 0.81 | 0.60 | 0.75 | 0.85 | 0.80 | 0.88 | 0.93 |
| Parahippocampal | 0.79 | 0.64 | 0.78 | 0.87 | 0.66 | 0.79 | 0.88 |
| Paracentral | 0.84 | 0.80 | 0.88 | 0.93 | 0.68 | 0.81 | 0.88 |
| Pars Opercularis | 0.92 | 0.87 | 0.92 | 0.95 | 0.85 | 0.91 | 0.95 |
| Pars Orbitalis | 0.78 | 0.61 | 0.76 | 0.85 | 0.67 | 0.80 | 0.88 |
| Pars Triangularis | 0.88 | 0.79 | 0.87 | 0.93 | 0.82 | 0.89 | 0.94 |
| Pericalcarine | 0.91 | 0.89 | 0.94 | 0.96 | 0.81 | 0.89 | 0.94 |
| Postcentral | 0.93 | 0.86 | 0.92 | 0.95 | 0.89 | 0.93 | 0.96 |
| Posterior Cingulate | 0.82 | 0.71 | 0.82 | 0.89 | 0.71 | 0.82 | 0.89 |
| Precentral | 0.95 | 0.90 | 0.94 | 0.97 | 0.92 | 0.95 | 0.97 |
| Precuneus | 0.89 | 0.84 | 0.90 | 0.94 | 0.79 | 0.87 | 0.93 |
| Rostral Anterior Cingulate | 0.80 | 0.68 | 0.81 | 0.89 | 0.67 | 0.80 | 0.88 |
| Rostral Middle Frontal | 0.90 | 0.84 | 0.90 | 0.94 | 0.83 | 0.90 | 0.94 |
| Superior Frontal | 0.88 | 0.82 | 0.89 | 0.94 | 0.78 | 0.87 | 0.92 |
| Superior Parietal | 0.89 | 0.86 | 0.92 | 0.95 | 0.77 | 0.86 | 0.92 |
| Superior Temporal | 0.93 | 0.89 | 0.94 | 0.96 | 0.86 | 0.92 | 0.95 |
| Supramarginal | 0.91 | 0.82 | 0.89 | 0.94 | 0.87 | 0.93 | 0.96 |
| Frontal Pole | 0.64 | 0.41 | 0.62 | 0.76 | 0.48 | 0.66 | 0.79 |
| Temporal Pole | 0.66 | 0.43 | 0.63 | 0.77 | 0.50 | 0.68 | 0.81 |
| Transverse Temporal | 0.92 | 0.87 | 0.92 | 0.96 | 0.85 | 0.91 | 0.95 |
| Insula | 0.85 | 0.77 | 0.86 | 0.92 | 0.73 | 0.84 | 0.91 |

**Supplement Table 5. White matter volume reliability**

| ROI | Hemisphere | 2.5% CI | ICC | 97.5% CI | Classification |
| --- | --- | --- | --- | --- | --- |
| Anterior Thalamic Radiation | lh | 0.96 | 0.98 | 0.99 | Excellent |
| Anterior Thalamic Radiation | rh | 0.97 | 0.98 | 0.99 | Excellent |
| Cingulum Cingulate Gyrus | lh | 0.99 | 0.99 | 1.00 | Excellent |
| Cingulum Cingulate Gyrus | rh | 0.98 | 0.99 | 0.99 | Excellent |
| Cingulum Hippocampus | lh | 0.93 | 0.96 | 0.98 | Excellent |
| Cingulum Hippocampus | rh | 0.95 | 0.97 | 0.98 | Excellent |
| Corticospinal Tract | lh | 0.96 | 0.98 | 0.99 | Excellent |
| Corticospinal Tract | rh | 0.97 | 0.98 | 0.99 | Excellent |
| Forceps Major | both | 0.97 | 0.98 | 0.99 | Excellent |
| Forceps Minor | both | 0.95 | 0.97 | 0.98 | Excellent |
| Inferior Fronto-Occipital Fasciculus | lh | 0.95 | 0.97 | 0.98 | Excellent |
| Inferior Fronto-Occipital Fasciculus | rh | 0.96 | 0.98 | 0.99 | Excellent |
| Inferior Longitudinal Fasciculus | lh | 0.95 | 0.97 | 0.98 | Excellent |
| Inferior Longitudinal Fasciculus | rh | 0.97 | 0.98 | 0.99 | Excellent |
| Superior Longitudinal Fasciculus Temporal | lh | 0.98 | 0.99 | 0.99 | Excellent |
| Superior Longitudinal Fasciculus Temporal | rh | 0.97 | 0.98 | 0.99 | Excellent |
| Superior Longitudinal Fasciculus | lh | 0.98 | 0.99 | 0.99 | Excellent |
| Superior Longitudinal Fasciculus | rh | 0.99 | 0.99 | 1.00 | Excellent |
| Uncinate Fasciculus | lh | 0.97 | 0.98 | 0.99 | Excellent |
| Uncinate Fasciculus | rh | 0.96 | 0.97 | 0.99 | Excellent |

**Supplement Table 6. Fractional anisotropy (FA) reliability**

| ROI | Hemisphere | 2.5% CI | ICC | 97.5% CI | Classification |
| --- | --- | --- | --- | --- | --- |
| Anterior Thalamic Radiation | lh | 0.73 | 0.84 | 0.90 | Excellent |
| Anterior Thalamic Radiation | rh | 0.73 | 0.84 | 0.90 | Excellent |
| Cingulum Cingulate Gyrus | lh | 0.81 | 0.88 | 0.93 | Excellent |
| Cingulum Cingulate Gyrus | rh | 0.69 | 0.81 | 0.89 | Excellent |
| Cingulum Hippocampus | lh | 0.88 | 0.93 | 0.96 | Excellent |
| Cingulum Hippocampus | rh | 0.72 | 0.83 | 0.90 | Excellent |
| Corticospinal Tract | lh | 0.74 | 0.85 | 0.91 | Excellent |
| Corticospinal Tract | rh | 0.69 | 0.81 | 0.89 | Excellent |
| Forceps Major | both | 0.90 | 0.94 | 0.97 | Excellent |
| Forceps Minor | both | 0.61 | 0.76 | 0.86 | Excellent |
| Inferior Fronto-Occipital Fasciculus | lh | 0.84 | 0.91 | 0.95 | Excellent |
| Inferior Fronto-Occipital Fasciculus | rh | 0.83 | 0.90 | 0.94 | Excellent |
| Inferior Longitudinal Fasciculus | lh | 0.91 | 0.95 | 0.97 | Excellent |
| Inferior Longitudinal Fasciculus | rh | 0.88 | 0.93 | 0.96 | Excellent |
| Superior Longitudinal Fasciculus Temporal | lh | 0.89 | 0.94 | 0.96 | Excellent |
| Superior Longitudinal Fasciculus Temporal | rh | 0.90 | 0.94 | 0.97 | Excellent |
| Superior Longitudinal Fasciculus | lh | 0.91 | 0.95 | 0.97 | Excellent |
| Superior Longitudinal Fasciculus | rh | 0.89 | 0.94 | 0.96 | Excellent |
| Uncinate Fasciculus | lh | 0.65 | 0.78 | 0.87 | Excellent |
| Uncinate Fasciculus | rh | 0.69 | 0.81 | 0.89 | Excellent |

**Supplement Table 7. Radial diffusivity (RD) reliability**

| ROI | Hemisphere | 2.5% CI | ICC | 97.5% CI | Classification |
| --- | --- | --- | --- | --- | --- |
| Anterior Thalamic Radiation | lh | 0.77 | 0.86 | 0.92 | Excellent |
| Anterior Thalamic Radiation | rh | 0.80 | 0.88 | 0.93 | Excellent |
| Cingulum Cingulate Gyrus | lh | 0.77 | 0.86 | 0.92 | Excellent |
| Cingulum Cingulate Gyrus | rh | 0.58 | 0.73 | 0.84 | Good |
| Cingulum Hippocampus | lh | 0.57 | 0.73 | 0.84 | Good |
| Cingulum Hippocampus | rh | 0.52 | 0.69 | 0.81 | Good |
| Corticospinal Tract | lh | 0.59 | 0.74 | 0.84 | Excellent |
| Corticospinal Tract | rh | 0.53 | 0.70 | 0.82 | Good |
| Forceps Major | both | 0.90 | 0.94 | 0.97 | Excellent |
| Forceps Minor | both | 0.87 | 0.92 | 0.96 | Excellent |
| Inferior Fronto-Occipital Fasciculus | lh | 0.79 | 0.87 | 0.93 | Excellent |
| Inferior Fronto-Occipital Fasciculus | rh | 0.80 | 0.88 | 0.93 | Excellent |
| Inferior Longitudinal Fasciculus | lh | 0.82 | 0.89 | 0.94 | Excellent |
| Inferior Longitudinal Fasciculus | rh | 0.67 | 0.80 | 0.88 | Excellent |
| Superior Longitudinal Fasciculus Temporal | lh | 0.85 | 0.91 | 0.95 | Excellent |
| Superior Longitudinal Fasciculus Temporal | rh | 0.85 | 0.91 | 0.95 | Excellent |
| Superior Longitudinal Fasciculus | lh | 0.87 | 0.92 | 0.96 | Excellent |
| Superior Longitudinal Fasciculus | rh | 0.83 | 0.90 | 0.94 | Excellent |
| Uncinate Fasciculus | lh | 0.70 | 0.82 | 0.89 | Excellent |
| Uncinate Fasciculus | rh | 0.63 | 0.77 | 0.86 | Excellent |

**Supplement Table 8. Mean diffusivity (MD) reliability**

| ROI | Hemisphere | 2.5% CI | ICC | 97.5% CI | Classification |
| --- | --- | --- | --- | --- | --- |
| Anterior Thalamic Radiation | lh | 0.79 | 0.87 | 0.93 | Excellent |
| Anterior Thalamic Radiation | rh | 0.81 | 0.89 | 0.93 | Excellent |
| Cingulum Cingulate Gyrus | lh | 0.69 | 0.81 | 0.89 | Excellent |
| Cingulum Cingulate Gyrus | rh | 0.53 | 0.70 | 0.82 | Good |
| Cingulum Hippocampus | lh | 0.46 | 0.65 | 0.79 | Good |
| Cingulum Hippocampus | rh | 0.43 | 0.63 | 0.77 | Good |
| Corticospinal Tract | lh | 0.52 | 0.69 | 0.81 | Good |
| Corticospinal Tract | rh | 0.48 | 0.66 | 0.79 | Good |
| Forceps Major | both | 0.89 | 0.93 | 0.96 | Excellent |
| Forceps Minor | both | 0.85 | 0.91 | 0.95 | Excellent |
| Inferior Fronto-Occipital Fasciculus | lh | 0.74 | 0.84 | 0.91 | Excellent |
| Inferior Fronto-Occipital Fasciculus | rh | 0.78 | 0.87 | 0.92 | Excellent |
| Inferior Longitudinal Fasciculus | lh | 0.75 | 0.85 | 0.91 | Excellent |
| Inferior Longitudinal Fasciculus | rh | 0.59 | 0.74 | 0.84 | Excellent |
| Superior Longitudinal Fasciculus Temporal | lh | 0.82 | 0.89 | 0.94 | Excellent |
| Superior Longitudinal Fasciculus Temporal | rh | 0.81 | 0.88 | 0.93 | Excellent |
| Superior Longitudinal Fasciculus | lh | 0.84 | 0.91 | 0.95 | Excellent |
| Superior Longitudinal Fasciculus | rh | 0.80 | 0.88 | 0.93 | Excellent |
| Uncinate Fasciculus | lh | 0.69 | 0.81 | 0.89 | Excellent |
| Uncinate Fasciculus | rh | 0.60 | 0.75 | 0.85 | Excellent |

**Supplement Table 9. Axial diffusivity (AD) reliability**

| ROI | Hemisphere | 2.5% CI | ICC | 97.5% CI | Classification |
| --- | --- | --- | --- | --- | --- |
| Anterior Thalamic Radiation | lh | 0.84 | 0.90 | 0.94 | Excellent |
| Anterior Thalamic Radiation | rh | 0.83 | 0.90 | 0.94 | Excellent |
| Cingulum Cingulate Gyrus | lh | 0.50 | 0.68 | 0.81 | Good |
| Cingulum Cingulate Gyrus | rh | 0.63 | 0.77 | 0.86 | Excellent |
| Cingulum Hippocampus | lh | 0.48 | 0.67 | 0.80 | Good |
| Cingulum Hippocampus | rh | 0.30 | 0.53 | 0.70 | Fair |
| Corticospinal Tract | lh | 0.43 | 0.63 | 0.77 | Good |
| Corticospinal Tract | rh | 0.47 | 0.66 | 0.79 | Good |
| Forceps Major | both | 0.88 | 0.93 | 0.96 | Excellent |
| Forceps Minor | both | 0.76 | 0.86 | 0.92 | Excellent |
| Inferior Fronto-Occipital Fasciculus | lh | 0.70 | 0.82 | 0.89 | Excellent |
| Inferior Fronto-Occipital Fasciculus | rh | 0.80 | 0.88 | 0.93 | Excellent |
| Inferior Longitudinal Fasciculus | lh | 0.63 | 0.77 | 0.86 | Excellent |
| Inferior Longitudinal Fasciculus | rh | 0.55 | 0.72 | 0.83 | Good |
| Superior Longitudinal Fasciculus Temporal | lh | 0.70 | 0.82 | 0.89 | Excellent |
| Superior Longitudinal Fasciculus Temporal | rh | 0.70 | 0.82 | 0.89 | Excellent |
| Superior Longitudinal Fasciculus | lh | 0.77 | 0.86 | 0.92 | Excellent |
| Superior Longitudinal Fasciculus | rh | 0.72 | 0.83 | 0.90 | Excellent |
| Uncinate Fasciculus | lh | 0.67 | 0.80 | 0.88 | Excellent |
| Uncinate Fasciculus | rh | 0.60 | 0.75 | 0.85 | Excellent |

**Supplement Table 10. Generalizability 14 months later**

| Measure | A/B ICC | C/D ICC |
| --- | --- | --- |
| Cortical Grey Matter Volume | **0.90**, 95% CI [0.84, 0.94] | **0.92,** 95% CI [0.83, 0.96] |
| Cortical Surface Area | **0.89**, 95% CI [0.82, 0.93]. | **0.92,** 95% CI [0.83, 0.96] |
| Cortical Thickness | **0.82**, 95% CI [0.71, 0.89]. | **0.80**, 95% CI [0.61, 0.91] |
| Cortical Folding (LGI) | **0.85,** 95% CI [0.75, 0.91] | **0.83,** 95% CI [0.66, 0.92] |

Scan-rescan ICC averaged across the Desikan atlas regions for common structural measures. A/B scans (N=100) from the main results. C/D ICC from a subset of scans (N=46) collected an average of 14 months after the A/B scans. ICC = intraclass correlation coefficient. LGI = local gyrification index. CI = confidence interval.

In addition to the 100 scans from 50 individuals reported on in the manuscript (timepoints A/B), we have collected an additional 46 scans from 23 individuals (timepoints C/D). These scans were collected an average of 14 months after the A/B scans and allow us to address the generalizability of the findings to the same scanner over a year later.

Overall, conducting the analysis in an identical way to the original sample, we again find excellent reliability in the subset of the same individuals scanned 14 months later. Furthermore, supplement table 10 shows the high consistency of the results.
